# Comparison of Genomic Profiling Data with Clinical Parameters: Implications for Breast Cancer Prognosis

**DOI:** 10.3390/cancers14174197

**Published:** 2022-08-30

**Authors:** José A. López-Ruiz, Jon A. Mieza, Ignacio Zabalza, María d. M. Vivanco

**Affiliations:** 1 Breast Imaging, PRETEIMAGEN Radiological Group, Manuel Allende 13, 48010 Bilbao, Spain; 2 Department of Oncological Gynecology, Cruces University Hospital, 48903 Barakaldo, Spain; 3 Pathology Department, Galdakao University Hospital, 48960 Galdakao, Spain; 4 Cancer Heterogeneity Lab, CIC bioGUNE, Basque Research and Technology Alliance, BRTA, Technological Park of Bizkaia, 48160 Derio, Spain

**Keywords:** breast cancer, IHC, TN, prognosis, Symphony, MammaPrint, genomic profile, pathological and molecular subtyping

## Abstract

**Simple Summary:**

Around 20 years ago, genomic profiling of breast carcinomas identified tumor subtypes with clinical implications and opened the door for a better understanding of breast cancer biology. The commercialization of multigene tests had a significant impact on clinical practice, and yet, controversy exists as to which methodology is best to inform the choice of therapy and existing recommendations are inconsistent and often driven by cost-effectiveness. Here we report data from a cohort of breast cancer patients in which pathological and molecular subtyping are directly compared in a clinical setting. The findings show that some patients with genomic low-risk tumors could receive unnecessary systemic therapy if only following the classical clinical parameters, while others could remain under-treated. This study suggests that to design precise treatment regimens for patients with early breast cancer, the conventional clinicopathological classification should be complemented with the robust prognostic information provided by molecular subtyping.

**Abstract:**

Precise prognosis is crucial for selection of adjuvant therapy in breast cancer. Molecular subtyping is increasingly used to complement immunohistochemical and pathological classification and to predict recurrence. This study compares both outcomes in a clinical setting. Molecular subtyping (MammaPrint^®^, TargetPrint^®^, and BluePrint^®^) and pathological classification data were compared in a cohort of 143 breast cancer patients. High risk clinical factors were defined by a value of the proliferation factor Ki67 equal or higher than 14% and/or high histological grade. The results from molecular classification were considered as reference. Core needle biopsies were found to be comparable to surgery samples for molecular classification. Discrepancies were found between molecular and pathological subtyping of the samples, including misclassification of HER2-positive tumors and the identification of a significant percentage of genomic high risk T1N0 tumors. In addition, 20% of clinical low-risk tumors showed genomic high risk, while clinical high-risk samples included 42% of cases with genomic low risk. According to pathological subtyping, a considerable number of breast cancer patients would not receive the appropriate systemic therapy. Our findings support the need to determine the molecular subtype of invasive breast tumors to improve breast cancer management.

## 1. Introduction

Breast cancer is a heterogeneous disease that, despite advances in basic and clinical research, continues to rise and remains the leading cause of death from cancer in women worldwide [1]. Molecular, cellular, and functional complexity within a tumor (intra-tumor heterogeneity), as well as diversity among tumors from different patients (inter-tumor heterogeneity), all contribute to confound diagnosis, challenge therapy, and promote the development of resistance. To identify the best possible breast cancer management practices, it is essential to use suitable predictive and prognostic tools to guide decision making and selection of treatments. Traditionally, breast cancers are classified according to histology, tumor size, lymph node status, histological grade, and the biomarkers ER (estrogen receptor), PR (progesterone receptor), and human epidermal growth factor receptor 2 (HER2) status, which are universally recommended [2]. Based on this information, clinicians select appropriate treatments, including radiotherapy, chemotherapy, hormone treatment, or antibodies targeting HER2.

Identification of the molecular subtypes of breast tumors based on their molecular profiles and their consequent association with clinical outcomes [3] provided new opportunities for tumor classification and prognostic tools. Over the last few years, several commercial multigene assays were developed for predicting outcome in patients with primary breast cancer. The characterization and commercialization of platforms, including multigene tests, contributed to improve predictive and prognostic tools for breast cancer management, although it remains to be demonstrated whether routine use of gene signature tests results in a better outcome for all patients [2,4,5,6,7].

Despite great advances in biomarker development, their routine application in the clinic is limited by their considerable cost. The standard prognostic biomarkers accepted in the clinic include expression of ER and PR for predicting response to endocrine therapy and amplification of HER2 for predicting benefit from anti-HER2 therapy. Further understanding of breast cancer heterogeneity raised increasing concerns that the well-established clinical and histological parameters may be too limited to portrait the diversity of breast cancer behavior in the clinic, and thus they would not address the need for personalized treatments. The limitations of the classical histological subtypes and the tumor-node-metastasis (TNM) staging system were questioned for some time, as they appear to be less accurate prognostic indicators to guide therapeutic decisions for breast cancer patients [8,9,10,11]. In addition, analytical discrepancies among different pathology laboratories were described, particularly for Ki67, which is used together with PR for luminal subtype identification, due to problems with its measurement and lack of standardization [12], highlighting the problem of subjectivity in marker quantification. The use of gene expression profiling in daily clinical practice may be challenging from an economical and practical point of view, which led to the examination of the potential of classical immunohistochemical (IHC) methods as an alternative for determining the molecular classification of invasive breast cancers. However, several reports revealed the flaws and ambiguities when attempting to classify tumors in molecular subtypes based on IHC surrogates [13]. For example, comparisons between the results obtained with the platform Symphony^TM^ (Agendia, Amsterdam, The Netherlands) and those from immunohistochemical analyses show discrepancies in the assignation of the molecular subtype [6]. These observations led to the proposal of a centralized pathological review [14], which could be useful, but not very practical. 

Findings in the prospective study MINDACT illustrated the value of the 70-gene signature test (MammaPrint) to predict clinical outcome and identify women that might not benefit from chemotherapy, highlighting the limitations of the clinical prognostic evaluation, when compared with genomic profiling [5]. Confirming these observations, a recent follow-up study of close to 9 years shows excellent distant metastasis-free survival for patients with high clinical risk and low genomic risk receiving no chemotherapy (endocrine therapy alone) [15]. In the same trial, further de-escalation of treatment is supported by the outcome of patients with an ultralow-risk 70-gene signature, with 8-year breast cancer specific survival above 99% and a distant metastasis-free interval rate of 97% [16]. These findings convincingly establish the predictive and prognostic information provided by these genetic platforms in terms of disease-free and overall survival. Therefore, the availability of multigene tests is shifting the paradigm of prognosis and treatment by providing additional personalized information about the molecular subtype of the cancer and assisting clinicians in complex therapy decisions.

Here, we present our experience in the determination of the genomic profile of a cohort of 143 consecutive breast tumors and the comparison with established clinical risk factors. To this end, the genomic test Symphony^TM^ Suite (MammaPrint^®^, BluePrint^®^, and TargetPrint^®^, Agendia, Amsterdam, The Netherlands) was used. MammaPrint uses a 70-gene expression signature to provide definitive high-risk or low-risk information about early-stage breast cancer recurrence in 5 years to determine if a patient is a candidate for chemotherapy. BluePrint 80-gene test looks at a tumor gene expression to classify it into molecular subtypes (luminal, basal, HER2) to guide the choice of therapies. TargetPrint offers a quantitative assessment of ER, PR, and HER2 expression to determine the potential benefit of hormonal therapy and targeted therapies. Given the predictive/prognostic power of the platforms described above, we consider the results obtained using the classification from the genomic tests as the gold standard in all correlative studies.

This study demonstrates the existence of significant discrepancies between molecular and pathological classification and, therefore, the risk for patients that, as a result, could be either over- or under-treated according to traditional clinical criteria.

## 2. Materials and Methods

### 2.1. Patients

A cohort of 143 consecutive patients with invasive breast carcinomas, between 36 and 82 years old, and collected by our multidisciplinary group was analyzed using the conventional pathological–clinical parameters and multigene tests in parallel. The protocol is indicated in Figure S1. Briefly, 2–3 core needle biopsies (CNB) of the breast were taken with a 12 G–14 G needle from a suspicious radiologic finding. Pathological analysis determined histological type, surgical locoregional status (according to the TNM system), histological grade, and immunohistochemical analyses of ER, PR, HER2, and Ki67 (including FISH/SISH when required). When the percentage of tumor cells was below 30% (minimum, as indicated by supplier), CNB needed to be repeated or the surgical specimen was used for determination of the genomic profile. When the tumor cell content was equal or above 30%, the samples were ready to be tested.

To determine the genetic profile, the platform Symphony^TM^ was used: MammaPrint^®^, a gene expression test with 70 genes to determine the risk of recurrence (available in 141 cases) TargetPrint^®^, which quantifies ER, PR, and HER2 status by measuring mRNA (available in 48 cases), and BluePrint^®^, an 80-gene test to identify the molecular subtype of the tumor (Luminal A, Luminal B, HER2, or basal-like) (available in 135 cases). The tumor samples analyzed included 47 CNB and 96 surgical specimens. The genomic profiles were classified as genomic high risk or low risk of recurrence or metastatic disease.

When the samples available included both biopsy and surgical specimens and a discrepancy was observed between them, whether IHQ and/or histological grade determinations, the result considered was that obtained from the surgical specimen.

Values of the proliferation factor Ki67 below 14% were considered negative, while those equal or higher than 14% were considered positive. The presence of high histological grade (G3) and/or Ki67 equal or higher than 14% were considered high clinical risk factors. In the clinical setting, potential HER2 enrichment was determined by IHC or FISH/SISH, if required.

The predictive/prognostic results obtained using the platform Symphony^TM^ are considered as the gold standard in all correlative studies.

### 2.2. Statistical Analysis

Chi-square tests for independence were used to examine the association between the clinical and genomic risk variables, where a two-sided *p* value < 0.05 was considered significant. The kappa statistic was used to determine the strength of the association/agreement between clinical and genomic variables. In kappa, complete agreement is represented by a value of 1.0 and only by chance by a value of zero. Although there is not a consensus standard criterion for kappa value that indicates adequate agreement, the following agreement measures for categorical data were proposed: kappa \0.00 represents poor agreement, 0.00–0.20 slight, 0.21–0.40 fair, 0.41–0.60 moderate, 0.61–0.80 substantial and 0.81–1.00 almost perfect agreement [17]. Calculations were done using IBM SPSS statistical software (IBM Corp, Version 26.0. Armonk, NY, USA) and VassarStats website for statistical computation.

## 3. Results

### 3.1. Similar Distribution of Genomic Risk Independently of Sampling Method or Age

**Figure 1:**
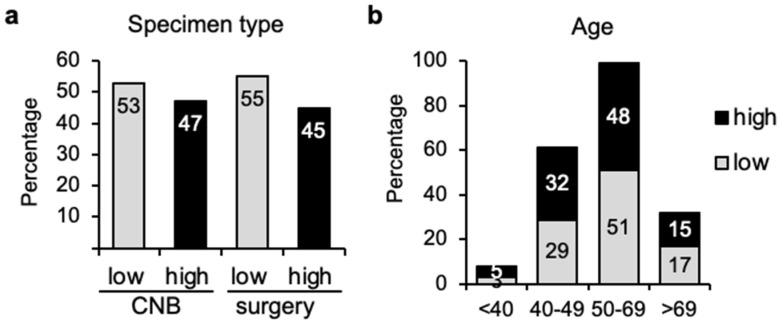
Distribution of breast cancers in genomic low or high risk depending on type of specimen or patient age. (**a**) Percentage of tumor samples (*n* = 143) obtained by core needle biopsy (CNB) (47 cases) or surgery (96 cases) that present genomic low or high risk according to MammaPrint genomic profiling. (**b**) 70-gene signature proportions (low or high risk) among patients of age as indicated.

The 70-gene signature MammaPrint was used in a cohort of 143 breast tumors to determine their risk of recurrence. Over half (55%) of the samples (78 cases) presented genomic low risk prediction and 45% of them were genomic high risk (65 cases). The tumor samples analyzed were either core needle biopsies (CNB) (47 cases) or surgical specimens (96 cases). The distribution of genomic low (53% vs. 55%) or high (47% vs. 45%) risk cases was remarkedly alike, independently of the method used for obtaining the tissue sample, CNB vs. surgery, respectively (Figure 1a), suggesting that CNB samples can be apt for genomic profiling, independently of genomic risk status.

Analysis of the age distribution showed that incidence was highest in the 50–69 age range, with only a modest increase in low-risk tumors in patients older than 50 (51% low- and 48% high-risk for the 50–69 group and 17% low- vs. 15% high-risk for the above 69 group) (Figure 1b). On the contrary, the percentage of high-risk patients was slightly higher in patients younger than 50 years (29% low- versus 32% high-risk for the 40–49 group). There were only five patients younger than 40 years old in the cohort and thus, the sample number was too small to make a conclusion about the significance of the distribution of genomic risk (2 low- vs. 3 high-risk cases).

### 3.2. Immunohistochemical Analysis and Genomic Risk Correlation

**Figure 2:**
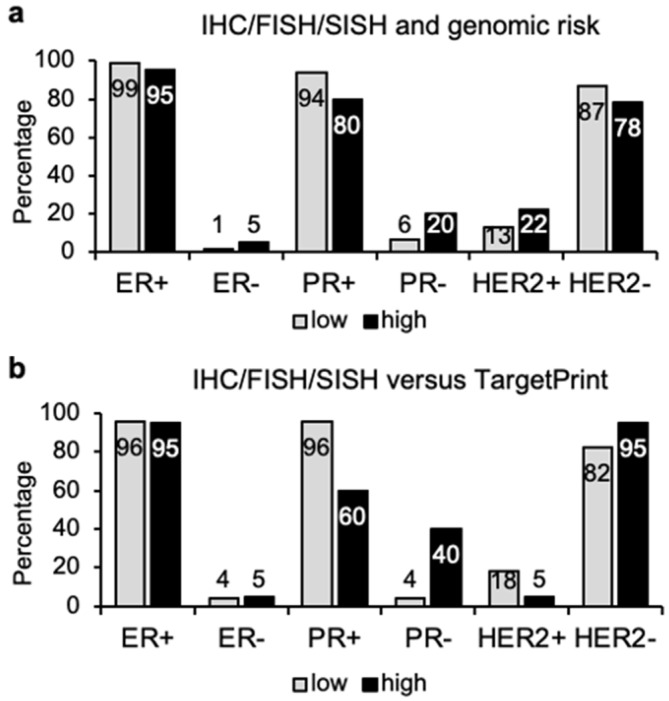
Distribution of breast cancers in genomic low or high risk depending on marker expression. (**a**) Proportion of tumor samples (*n* = 143) with genomic low (78 cases) or high risk (65 cases) that presented expression or lack of the indicated individual markers (estrogen receptor, ER, progesterone receptor, PR, and overexpression of HER2) according to immunohistochemistry (IHC), fluorescence in situ hybridization (FISH) or silver-enhanced in situ hybridization (SISH). (**b**) Proportion of tumor samples (*n* = 48) classified by TargetPrint as genomic low (28 cases) or high risk (20 cases) that presented expression of the markers as in (**a**).

Comparison of immunohistochemical analysis of the 143 tumors with their multigene tests showed that tumors classified as genomic low risk (78 cases), also included 10 (13%) HER2+ tumors, as well as 1 ER- and 5 (6%) PR- cases (Figure 2a). In contrast, genomic high risk (65 cases) cases also contained a high percentage of ER+/PR+ tumors (Figure 2a). Analysis of these markers using CNB (Figure S2a) or surgical (Figure S2b) samples did not significantly affect these observations, which again supports the use of CNB samples for this type of analysis.

Quantification of ER, PR, and HER2 status with TargetPrint was obtained for 48 cases, 28 low risk and 20 high-risk tumors (Figure 2b and Table 1). Comparative analysis between TargetPrint and IHC/FISH/SISH assessment showed almost perfect agreement for ER expression (Kappa statistic = 0.985, *p* < 0.001). Although PR expression displayed a certain level of discrepancy, with five cases (11%) that were considered positive by IHC analysis, but PR- by TargetPrint (Table 1), the Kappa statistic was still very good (0.928, *p* < 0.001). Inconsistencies were also observed for HER2 overexpression, which was found in eight cases by IHC and FISH, but only four of them (50%) were confirmed by TargetPrint (Kappa statistic = 0.813, *p* < 0.001). Most cases (40/48) were HER2- by clinical assessment and yet, two of them (5%) were identified as positive by TargetPrint. If we consider the HER2 determinations obtained by TargetPrint, together with those obtained by BluePrint, we have 137 cases for comparative analysis (Table 1 and Table S1), which showed high agreement for HER2- cases (97%), but low agreement in HER2+ cases (52%). These findings highlight some lack of correlation between genomic risk and clinical markers, particularly for PR and HER2.

### 3.3. Molecular Subtype Correlation

BluePrint data were available for 135 cases (Table 1). A total of 44 breast tumors were classified as Luminal A by IHC/FISH, but 20% of them disagreed with BluePrint data, which included 1 basal (2%) and 8 Luminal B (18%) tumors. In contrast, within the 88 tumors identified as Luminal B by surrogate markers, 40% of them were Luminal A, 6% were HER2+ (5 cases) and one of them was basal. Of the two cases classified as basal, only one of them was confirmed to be basal by BluePrint, while the other was identified as Luminal A. Statistical analysis showed a significant association between the classification of tumors by BluePrint compared with IHC/FISH (χ(12) = 202.861, *p* < 0.001), however, the Kappa statistic (0.529, *p* < 0.001) indicated only a moderate agreement, reflecting the observation that a considerable number of tumors were classified as belonging to a different molecular subtype depending on the classification method used. The main differences reside in the classification of tumors as Luminal B, which were particularly overestimated using surrogate markers. In addition, it should be noted that there are considerable differences in the classification of HER2 tumors, with approximately only half of the tumors classified as HER2+ by IHC/FISH/SISH being recognized as positive by the genomic classification, while the agreement in HER2- tumors is almost total (Table 1). These notable disagreements highlight the limited value of IHC/FISH evaluation to determine the molecular subtype of some breast tumors.

### 3.4. Clinical Risk vs. Genomic Risk

Comparative analysis of genomic (MammaPrint platform) and clinical risk parameters of 141 tumor samples (Table 1) showed that 95 cases (67%) with clinical high risk included 40 cases (42%) with genomic low risk, according to MammaPrint. On the other hand, 9 (20%) of the 46 tumors with clinical low risk were identified as genomic high risk by MammaPrint. These findings show the considerable percentage of patients that would receive unnecessary systemic therapy when presenting genomic low-risk tumors if only following the classical clinical parameters, while on the other hand, other patients would remain under-treated.

### 3.5. Immunohistochemical Analysis and Genomic Risk Correlation

**Figure 3:**
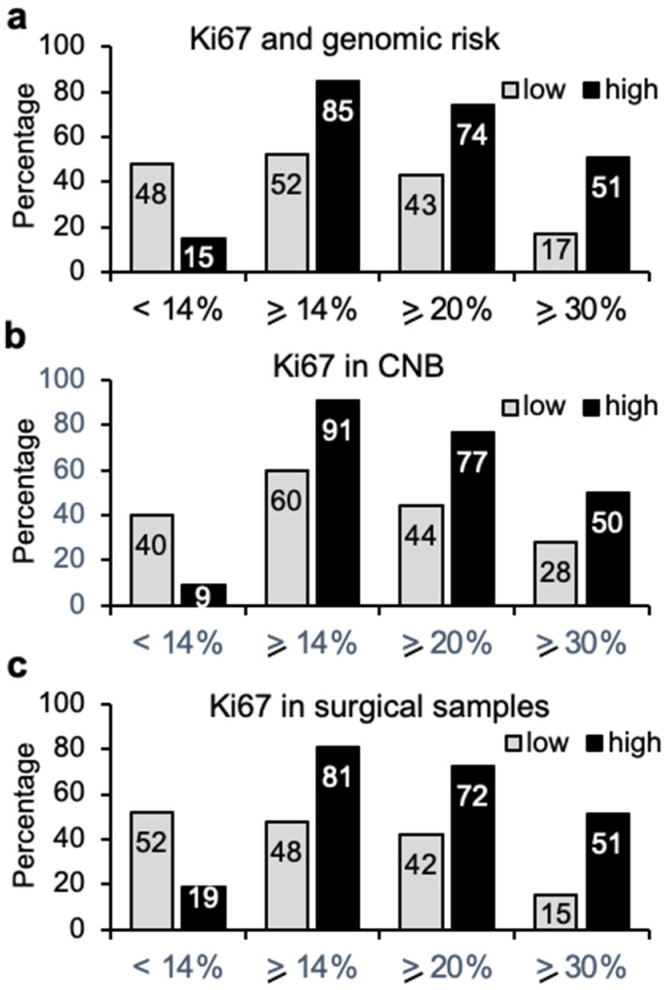
Proportions of 70-gene signature results according to Ki67 expression. (**a**) Proportion of tumor samples (*n* = 142) with genomic low (77 cases) or high risk (65 cases) with levels of Ki67 lower than 14% or equal/higher than 14%, 20%, or 30%. (**b**) Percentage of tumor samples obtained by core needle biopsy (CNB) (47 cases) that present genomic low (25 cases) or high (22 cases) risk according to Ki67 expression levels. (**c**) Percentage of samples obtained by surgery (95 cases) with genomic low (52 cases) or high (43 cases) risk according to Ki67 expression levels.

The value of the proliferation factor Ki67 as a marker for treatment decisions in breast cancer patients is a long-standing a matter of debate. Data for 142 tumor samples were available for analysis of Ki67 expression. Among the genomic low-risk tumors, 52% showed a Ki67 index equal or higher than 14% (Figure 3a), which is associated with clinical high risk. Of those, 33 (43%) and 15 (17%) cases showed a Ki67 index equal or higher than 20% and 30%, respectively. On the other hand, 15% of genomic high-risk tumors presented Ki67 lower than 14%, and 51% had Ki67 higher or equal than 30%. Independent analysis of Ki67 expression in CNB (47 cases, Figure 3b) and surgical specimen (95 cases, Figure 3c), showed only modest differences. CNB samples showed higher agreement between both types of assays for genomic high-risk tumors, while classification of surgery samples was more similar for genomic low-risk tumors.

### 3.6. Histological Grade and Genomic Risk Correlation

**Figure 4:**
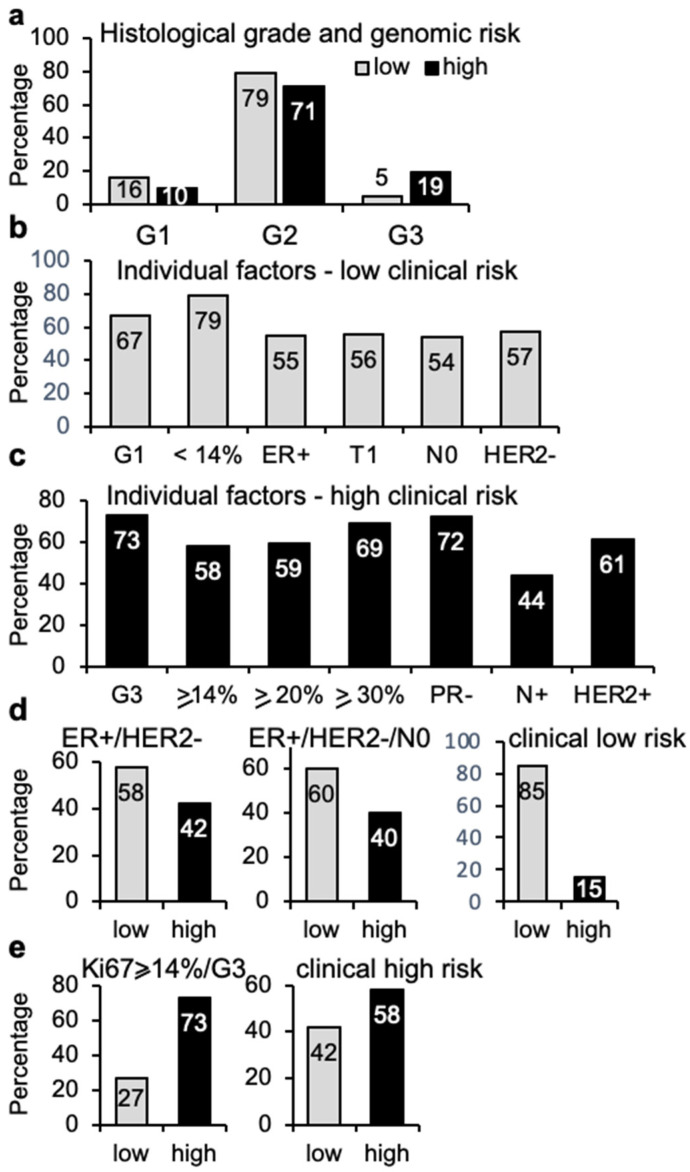
Distribution of 70-gene signature among various clinical markers. (**a**) Proportion of tumor samples (*n* = 136) with genomic low (77 cases) or high (59 cases) risk according to histological grade (G). (**b**) Distribution of samples with genomic low risk according to individual clinical low-risk parameters: G1 (18 cases), low Ki67 (47 cases), ER+ (139 cases), T1/N0 (88 cases), N0 (107 cases), and HER2- (119 cases). (**c**) Genomic high-risk samples according to clinical high-risk parameters: G3 (15 cases), high Ki67 (95 cases), PR- (18 cases), Node+ (36 cases), and HER2+ (23 cases). (**d**) Genomic profile of 115 ER+/HER2- tumors (left), including N0 (center, 83 cases) and low Ki67 (right, 35 cases). (**e**) Genomic profile of 13 G3 tumors with high Ki67 expression (left), and 43 tumors with high clinical risk (right).

The degree of differentiation of the tumor tissue is reflected in the histological grade, which is considered of prognostic value, with high tumor grade associated to high clinical risk. The histological grade was known for 136 cases (Figure 4a). G2 grade was the most common both in genomic low- (79%) and high-risk (71%) tumors. Notably, there were four high-grade tumors (5%) in the genomic low risk group. In contrast, genomic high-risk tumors included six cases with low G1 grade (10%). Although the numbers are low, these observations illustrate that the distribution of histological grade across tumors of low and high risk is not sufficient to serve as an individual prognostic factor.

Genomic risk was also compared with other individual clinical parameters, including grade, size, node implication, percentage of Ki67 cells, ER and PR expression, and HER2 overexpression to assess whether the use of genomic risk profiling would improve the positive predictive value (PPV) for breast cancer prognosis. The conventional low-risk parameters according to IHC data included: low histological grade (G1), Ki67 lower than 14%, ER positivity, T1 (tumor is 2cm across or less), N0 (no lymph nodes implicated) and lack of HER2 overexpression. In contrast, the clinical high-risk parameters included: high grade (G3), high Ki67, which was further divided into higher or equal to 14%, 20%, and 30%, lymph node involvement, PR negativity, and HER2 overexpression.

Analysis of classical low-risk parameters showed that Ki67 lower than 14% was the parameter that correlated the most with genomic low risk (79%), while only 54% of N0 and 55% of ER+ tumors were of genomic low risk (Figure 4b). On the other hand, analysis of individual high-risk parameters identified G3 (73%), PR negativity (72%), and Ki67 higher or equal to 30% (69%) as the factors with the highest association with high risk (Figure 4c). Interestingly, the absence or presence of cancer cells in the lymph nodes appeared to display the lowest association with genomic risk, with only 44% of cases with lymph node implication presenting high genomic risk (Figure 4c).

To clarify further the relationship between clinical risk factors and genomic risk, some further analyses were performed. Determination of ER+, HER2- (115 cases), which could be considered of clinical low risk, showed that 42% of them (48 cases) displayed genomic high risk (Figure 4d). Including the information about node spreading resulted in a cohort of 83 ER+/HER2-/N0 tumors, this is without cancer cells in the nearby nodes, which still contained 33 (40%) genomic high-risk cases (Figure 4d). Furthermore, adding the value of Ki67 lower or equal to 14% and low grade (35 tumors), still meant 15% of them were of genomic high risk (Figure 4d), despite their classification as tumors of low clinical risk. The group of 15 highly proliferative (Ki67 >/= 14%) and high-grade (G3) tumors included four cases (27%) with genomic low risk (Figure 4e). Finally, a combination of ER+/HER2-/N0 with high grade and Ki67 higher than 14% in 43 cases (high clinical risk) showed 42% of them were genomic low risk (Figure 4e). The six cases that were PR- and G3 were all genomic high-risk tumors. Together, these findings illustrate that among tumors considered of low risk by various clinical markers, a significant percentage of them are of high genomic risk.

### 3.7. TN Staging and Genomic Risk Correlation

**Figure 5:**
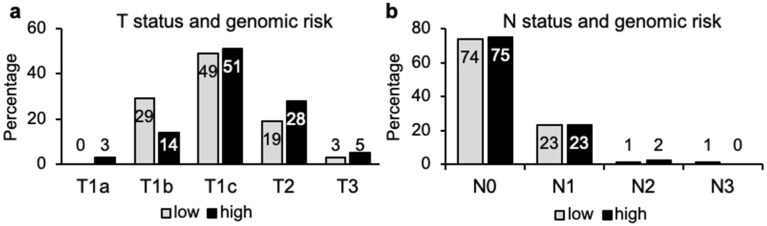
Comparison of 70-gene signature results with TN classification. (**a**) Proportion of tumor samples (*n* = 143) with genomic low (78 cases) or high risk (65 cases) according to their tumor size (T) or in (**b**) node involvement (N).

Within the cohort of 143 tumors, 68% of genomic high-risk (44/65) cases were T1 breast tumors (equal or less than 2 cm). On the other hand, 22% of genomic low-risk (17/78) cases were T2/T3 tumors (Figure 5a). Furthermore, the distribution of low and high genomic risk among N0 and N1 tumors was remarkably similar. In total, 75% of the genomic high risk (49/65) were N0 tumors (Figure 5b); there was only one N3 case, which showed low genomic risk. Interestingly, 39 samples (44%) were genomic high risk within the 88 T1N0 breast tumors examined. These findings suggest that TN staging shows limited prognostic value, since even early T1 N0 breast cancer does not represent a good prognosis in a considerable number of cases.

## 4. Discussion

This study illustrates the existing variability between current routine clinical and pathological prognostic and predictive factors and molecular classification to guide clinical decisions in breast cancer management. Although the number of tumors analyzed is relatively modest, the findings show that genomic profiling identifies a higher number of low-risk tumors (77 cases, 55% of total) than conventional clinical classification (46 cases, 33% of total), with the corresponding implications for therapy selection. Additionally, they reveal that a considerable percentage of small tumors without node implication, 44% of T1N0 cases in this cohort, can display genomic high risk. These findings support the limited predictive/prognostic value of the TNM system, as also shown by others [18], although it is still useful in the loco-regional control of the disease [19].

The distribution of cases with low or high genomic risk was similar according to age, as previously reported [20]. Genomic low or high-risk distributions were similar between samples tested from CNB versus surgical resection, suggesting that genomic testing on CNB can be reliably used to guide breast cancer management, thus accelerating prognostic evaluation. Similarly, the EndoPredict multigene test is shown to score comparably between core biopsies and surgical specimens [21]. On the other hand, others found that selection of samples with a high tumor percentage for gene expression profiling may lead to enrichment of high-grade tumors [22], although advances in the techniques used by commercial platforms reduced the minimum tumor composition of the tissue required, which can be as low as 30% [23].

Comparison of commercial multigene assays and clinicopathological subtypes for breast cancer already revealed some discrepancies, whether using PAM50/Prosigna, based on unsupervised clustering [24] or combined MammaPrint and BluePrint tests, which were developed during a supervised training [6]. In our study, we observed 35% (49/141) of cases whose clinical risk classification differed from the risk identified by molecular assessment. These differences were mostly due to the over-estimation of risk due to the factor Ki67 equal to or higher than 14%, but also in cases with values equal to or higher than 20% and 30%. The most common Ki67 value found (85% of cases) was equal or higher than 14% in tumors of high genomic risk. However, even among the genomic low-risk tumors, 52% of them had Ki67 higher than 14%, which is considered a clinical high-risk factor. Ki67 measurements are notorious for lacking inter-laboratory reproducibility [25] and recommendations over the years are evolving, including stratification for patients with intermediate (14 to 19%) or high (≥20%) Ki67 positivity combined with PR positivity as a surrogate of the molecular analysis for distinguishing Luminal A from Luminal B tumors [26,27]. A recent assessment by the International Ki67 in Breast Cancer Working Group confirmed substantial inter-observer/laboratory variability in the range of >5 to <30% and thus recommends that Ki67 analysis avoids this range to drive patient care and estimate prognosis [28]. A study of patients enrolled in the MINDACT trial shows that 18% is the optimal threshold for the best correlation between Ki67 and the results of MammaPrint [6], while 13.25% was the threshold identified by the PAM50 test to distinguish Luminal A and Luminal B tumors [29]. Our study still found that 15% of cases with low Ki67 values (<14%) were of high genomic risk, highlighting the need for careful use of this marker in clinical management of the disease.

Analysis of the immunohistochemical data for ER, PR, and HER2 did not reveal statistically significant differences between low and high genomic risk. Nevertheless, a marked increase in the percentage of PR-negative tumors in genomic high-risk tumors was found, in agreement with others that observed the association of ER-positive/PR-negative tumors with a higher risk of recurrence [30], particularly in the context of high proliferative tumors [31]. Similarly, our own work showed a significant increase in PR-negative tumors during the development of resistance to tamoxifen, accompanied by increased aggressiveness and cancer stem cell content [32,33]. Indeed, a recent meta-analysis of breast cancer recurrence over a 20-year period showed PR status as an independent prognostic factor during the first 5 years, but not thereafter [34]. Thus, the relevance of PR expression is becoming more apparent and the under-representation of PR negative tumors by immunohistochemistry analysis may be of clinical significance if the bad prognosis of a lack of PR is confirmed in further studies.

Pathological classification of HER2 by immunohistochemistry is complemented by fluorescence in situ hybridization or silver-enhanced in situ hybridization when confirmation is needed. However, 48% of the tumors considered as positive, were found to be negative by genomic testing (TargetPrint and BluePrint). In this cohort, although the numbers are low, the discrepancy detected between genomic and pathological classification of HER2-positive and basal tumors was higher than previously reported [6], which highlights the potential risk for both over- and under-treatment with anti-HER2 therapy.

Difficulties in the standardization of the assessment of Ki67 and lack of clear cut-off values contributed to the use of histological grade as a better surrogate marker for proliferative activity [35], and high grade (G3) represents a better association with tumor aggressiveness than the factor Ki67 [31], offering a high prognostic value. However, although is a very small number, four G3 tumors were found in the genomic low-risk group, in agreement with previous data reporting some luminal A tumors with grade 3 and low Ki67 [24]. On the other hand, the genomic high-risk group included a majority of tumors with intermediate histological grade. These findings illustrate the poor association between tumor grade and prognosis. Similarly, despite the association previously found between N0 tumors and a good clinical prognosis, defined as Ki67 lower than 14% and G1 histological grade [36], our data indicate the poor prognostic value of lack of node involvement.

The discrepancies found during this comparative study have significant consequences for the therapeutic treatments offered to patients. An elevated proportion of patients who presented high clinical risk (42%) displayed genomic low risk and, therefore, those patients would receive an aggressive systemic over-treatment. These findings agree with other reports, for example, in the MINDACT study, 46.2% of the patients at clinical high risk were classified by MammaPrint as genomic low risk [5], while a Dutch study reported that MammaPrint classified 52.6% of clinical high-risk tumors as genomic low risk [18], supporting de-escalation. Recent guidelines suggest the use of the 70-gene signature only for women at high clinical risk [37]. However, although the agreement of clinical low risk with genomic data were high, 20% of cases displayed genomic high risk, implying that these patients would not receive the required therapy, and thus possibly resulting in their under-treatment. The problem of under-treatment in breast cancer is less often discussed; however, it was found that one out of three patients with axillary node-negative early breast cancer could develop metastasis [19], which could be partly due to under-treatment.

The genomic platforms appear to be the best prognostic tools for invasive breast cancer management [38], and yet, there is not always strong consensus regarding which patients would benefit from them [37,39,40]. Some proposed criteria include ER-positive, HER2-negative, well-differentiated tumors (G1), without affected lymph nodes (N0), and low Ki67 [41]. However, our cohort of 107 N0 tumors only included thirteen cases that would comply with such criteria, three of them (23%) with genomic high risk. Other proposed criteria do not include the value of Ki67 among the required criteria [42], which increases the number of cases that would be recommended for genetic analysis. Similarly, genomic classification in clinically low-risk T1N0 ER-positive tumors to identify candidates for extended endocrine therapy, is recommended [43].

Previously, it was reported that women in the clinical low risk category did not benefit from chemotherapy regardless of MammaPrint genetic risk [40]. A recent update confirmed that omitting chemotherapy in clinical high/genomic low postmenopausal women continues to be safe, although this effect appears to be clearer in women younger than 50 years [15]. Furthermore, the use of the 70-gene signature to safely guide chemotherapy de-escalation in clinical high-risk patients can result in a more quality-adjusted life; in addition, it can also be cost-effective [44]. Similarly, integration of genomic information based on the 21-gene breast cancer assay and clinical risk stratification based on tumor size and histological grade was shown to provide prognostic information about recurrence, but not prediction of chemotherapy benefit [20]. On the other hand, a recently published consensus statement suggests the use of MammaPrint gene signature only for women at high clinical risk [37] and, although the grading of certainty is different depending on the guidelines used, economical concerns are discussed, as well as the fact that decreasing costs for the tests would support a more widespread use. The findings from this study suggest that to avoid under-treatment of some of these patients with low clinical risk, the conventional clinical information could be complemented with the robust prognostic information provided by molecular subtyping.

Nevertheless, genomic tests are not the solution for every situation from the predictive and prognostic point of view. Although we continue to learn about the biological behavior of breast tumors (and genomic profiling proves fundamental for this), many unknowns remain. Both inter-patient and intra-tumor heterogeneity (including the presence of cells with properties of stem cells) continue to be a challenge for breast cancer management, and therapy failure in a significant number of patients remains a serious clinical problem [34]. Furthermore, metastatic breast cancers display an increase in mutational burden and clonal diversity compared to early breast cancers [45], thus adding further complexity and defying current treatments.

## 5. Conclusions

In conclusion, full agreement does not exist between BluePrint, TargetPrint, and determinations by IHC and/or FISH, nor in the assignment of the molecular subtypes, neither for hormonal receptors or HER2. The major discrepancies are observed in the allocation of the molecular subtype, due principally to the presence of a Ki67 factor equal to/greater than 14%, for which its prognostic importance (even considering a value equal to/greater than 20%) is probably overrated. The discrepancies, regarding the molecular subtype, are observed with greater frequency among the low-risk tumors. While histological grade does not appear to be an individual prognostic factor, high-grade (G3) and PR-negative tumors were all genomic high risk and presented the highest positive predictive value, although the small numbers (*n* = 6) weaken this conclusion. Finally, in compliance with the criteria of St. Gallen (2017), it is not possible to unequivocally differentiate patients with high or low genomic risk based on the usual clinical risk criteria. Thus, it is probably necessary to resort to genomic tests in all cases of infiltrating breast cancer.

## Figures and Tables

**Table 1:** Breast cancer classification according to pathological subtyping or molecular classification using the Symphony platform. The number of cases for each type, as well as the percentage of agreement between both methods is indicated.

IHC/FISH/SISH	Nr Cases	TargetPrint	Nr Agree (%)
ER+	47	ER+	46 (98%)
		ER−	1
ER−	1	ER−	1 (100%)
PR+	44	PR+	39 (89%)
		PR−	5
PR−	4	PR−	4 (100%)
HER2+	8	HER2+	4 (50%)
		HER2−	4
HER2−	40	HER2−	38 (95%)
		HER2+	2
**IHC/FISH/SISH**	**Nr Cases (%)**	**BluePrint**	**Nr Agree (%)**
LuminalA	44 (33%)	Luminal A	35 (80%)
		Luminal B	8 (18%)
		Basal	1 (2%)
Luminal B	88 (65%)	Luminal B	47 (53%)
		Luminal A	35 (40%)
		HER2	5 (6%)
		Basal	1 (1%)
Basal	3 (2%)	Basal	2 (67%)
		Luminal A	1 (33%)
HER2+	0		
**IHC/FISH/SISH**	**Nr Cases (%)**	**TargetPrint/BluePrint**	**Nr Agree (%)**
HER2+	23 (16%)	HER2+	12 (52%)
		HER2−	11 (48%)
HER2−	114	HER2−	111 (97%)
		HER2+	3 (3%)
**Clinical risk**	**Nr Cases (%)**	**MammaPrint**	**Nr Agree (%)**
High	95 (67%)	High	55 (58%)
		Low	40 (42%)
Low	46 (33%)	Low	37 (80%)
		High	9 (20%)

## Data Availability

The datasets used and analyzed during the current study are available from the corresponding authors on reasonable request.

## Supplementary Materials

The following supporting information can be downloaded at: https://www.mdpi.com/article/10.3390/cancers14174197/s1, Figure S1: Protocol for tumor sample collection and analysis; Figure S2: Distribution of breast cancers in genomic low or high risk depending on marker expression; Table S1: HER2 expression analysis.
