# Supplementary material for: Comparison of Genomic Profiling Data with Clinical Parameters: Implications for Breast Cancer Prognosis
Source: Cancers (Basel). 2022 Aug 30;14(17):4197. doi: 10.3390/cancers14174197 (PMC9454811; doi:10.3390/cancers14174197)
Supplement: Supplementary file 1 [file cancers-14-04197-s001.zip › cancers-1878472-supplementary.pdf]

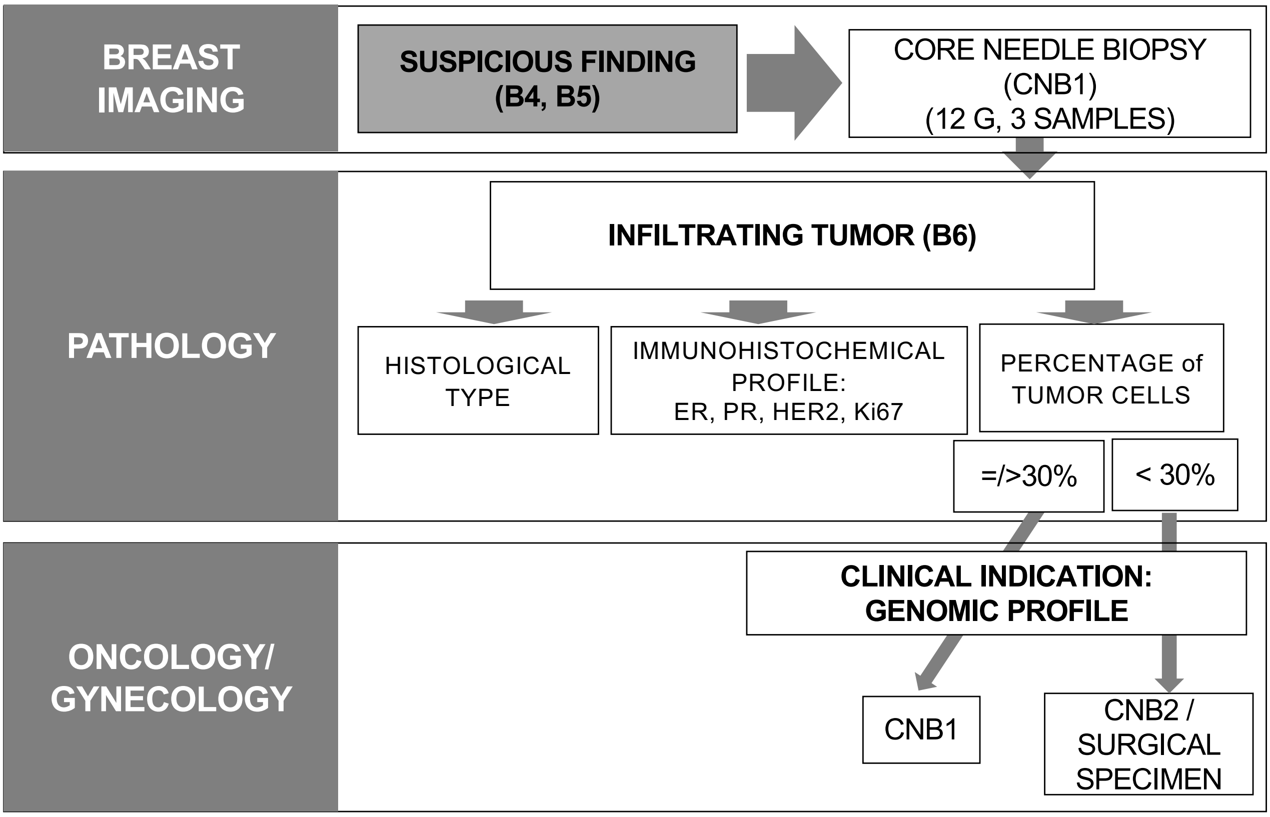

Supplementary Figure S1. Protocol for tumor sample collection and analysis.

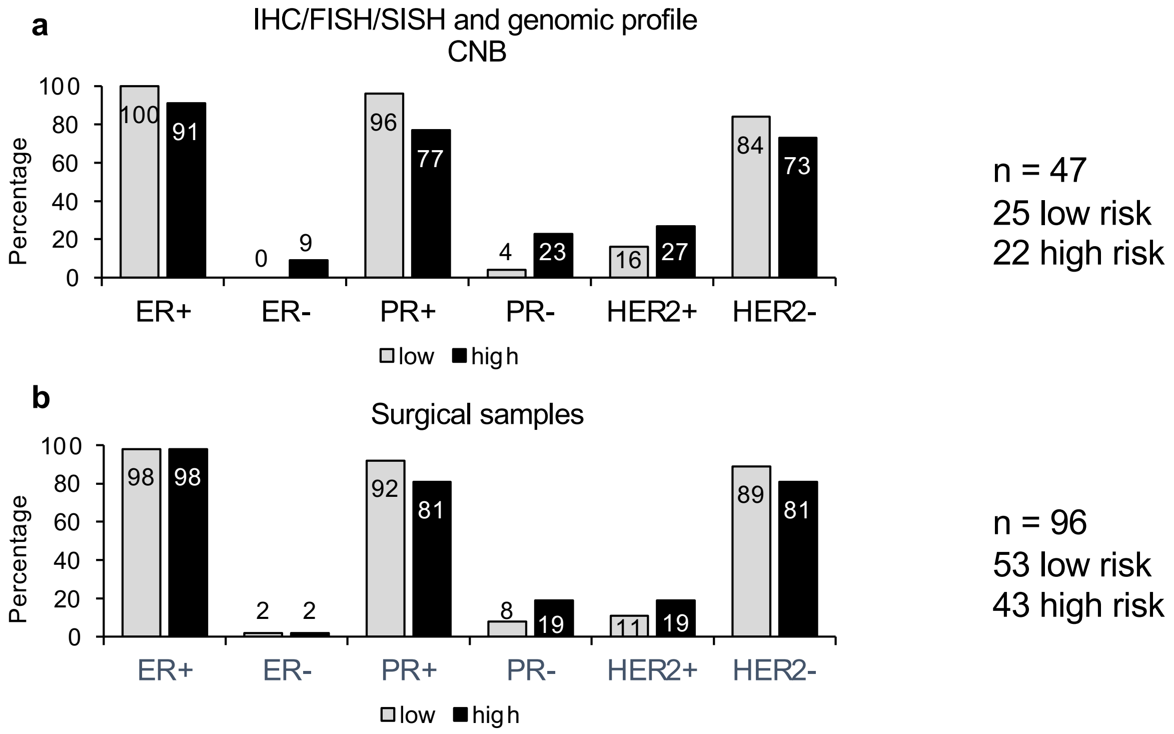

Supplementary Figure S2. Distribution of breast cancers in genomic low or high risk depending on marker expression. (**a**) Proportion of tumor samples obtained by core needle biopsy (n = 47) with genomic low (25 cases) or high risk (22 cases) that presented expression or lack of the indicated individual markers (estrogen receptor, ER, progesterone receptor, PR, overexpression of HER2) according to immunohistochemistry (IHC), fluorescence *in situ* hybridisation (FISH) or silver-enhanced *in situ* hybridization (SISH). (**b**)Proportion of tumour samples obtained by surgery (n = 96) classified by TargetPrint as genomic low (53 cases) or high risk (43 cases) that presented expression of the markers as in **a**.

IHC/FISH/SISH	CASES (n=137)	TARGETPRINT® (n=48)		BLUEPRINT® (n= 89)		TARGETPRINT® / BLUEPRINT® (n= 137)		AGREEMENT (%)
		HER2 +	HER2 -	HER2 +	HER2 -	HER2 +	HER2 -	
**HER 2 +**	23	4	4	8	7	12	11	**52.17%**
**HER 2 -**	114	2	38	1	73	3	111	**97.37%**
**TOTALS**	137	6	42	9	80	15	122	

Supplementary Table S1. HER2 expression analysis. Comparative analysis between clinical assessment of HER2 overexpression/amplification using immunohistochemistry (IHC), fluorescence in situ hybridization (FISH) or silver in situ hybridization (SISH) in a total of 137 tumor samples, using the microarray-based test TargetPrint in 48 samples and BluePrint in 89 samples. The agreement between clinical surrogate and genomic profile data is indicated as percentage (%).
